# Characteristics, risk behaviors and factors associated with abortion among female entertainment workers in Cambodia

**DOI:** 10.1186/s12978-015-0075-y

**Published:** 2015-09-04

**Authors:** Heng Sopheab, Sovannary Tuot, Chhorvann Chhea, Pamina Gorbach

**Affiliations:** School of Public Health at the National Institute of Public Health, Cambodia, # 2, Street 289, Tuol Kork District, Phnom Penh, Cambodia; Research Department, KHANA, Phnom Penh, Cambodia; Department of Epidemiology, Fielding School of Public Health, University of California, Los Angeles, USA

## Abstract

**Background:**

Linkages between HIV and reproductive health (RH) among female entertainment workers (EWs) have not been addressed well in most developing countries. In Cambodia, there has been considerable research on HIV epidemiology among EWs. However, there have been limited studies on RH and specifically factors related to abortion. We examine socio-behavioral characteristics, and RH practices as determinants of abortion among Cambodian EWs.

**Methods:**

A survey was conducted in Siem Reap and Battambang and Phnom Penh provinces/city among EWs in September 2012. Trained female interviewers administered the survey to 595 EWs. Two-stage cluster sampling was used to select EWs. Bivariate associations were examined using chi-squares; univariate and multivariate logistic regression were used to assess factors independently associated with reporting having at least one abortion while working as an EW.

**Results:**

Three-quarters (75 %) of EWs were sexually active, of which nearly one third reported at least one abortion while working as an EW. About 40 % of EWs reported recent an abortion in the past six months. Contraceptive use in the past year was low. Factors found to be independently associated with reporting a recent abortion included: ages 25–29 (OR = 2.2, 95 % CI: 1.2–4.0), living with spouse/cohabitated partner (OR = 2.2, 95 % CI: 1.1–4.2), longer duration of entertainment work (OR = 4.8, 95 % CI: 2.5–9.2), higher number of partners (OR = 4.4, 95 % CI: 2.2–8.7) and being a karaoke worker (OR = 2.2, 95 % CI: 1.1–4.4).

**Conclusion:**

This study highlights a high proportion of EWs reporting abortion. While HIV vulnerability of EWs has been clearly established, broader RH needs have gone largely unrecognized and not prioritized. Though HIV/RH integrated initiatives have been introduced by the HIV program, challenges for EWs with multiple risks from professional and partners are profound. Therefore, there is an urgent need for practical programmatic approaches to help address their RH needs.

## Introduction

Abortion is a major public health concern in many parts of the world, particularly in developing countries where access to safe services is limited [1]. Globally, each year, an estimated 42 million abortions, both safe and unsafe, are performed [2]. More than half of abortions occur outside a legal system and are considered unsafe because they are mostly performed by unskilled and poorly trained providers or in settings that do not meet minimal medical standards. In developing countries, two in five abortions occur among women aged under 25 [1, 3, 4]. Globally unsafe abortions accounted for 13 % of maternal related death, in which most of the cases could be preventable by access to appropriate safe abortion services [5].

In Cambodia, 5 % of women aged 15–49 reported having had an abortion in the past five years and 43 % of the abortions took place outside health facilities [6]. Although abortion is legal in Cambodia before 12 weeks of pregnancy, there is a lack of trust in those available through the public health system because services are limited and facilities understaffed with compromised quality of care [7]. Among female entertainment workers, abortions and unintended pregnancies are highly prevalent, ranging from 25 % to 31 % among different types of entertainment workers [8–12]. Studies in different parts of the world **−** Russia, Colombia, Hong Kong, and Thailand **−** reported that more than 50 % of the female sex workers reported having experienced at least one abortion in their life time. The risk of having an abortion has been reported to be associated with increased age, duration of sex work and number of partners [9, 13–15].

In Cambodia, after the implementation of the law on women trafficking and exploitation in 2008 there was a crackdown on brothel-based sex establishments. In this context a new term, “female entertainment workers (EWs)”, began to be used to refer to both direct female sex workers (those based in brothels) and indirect female sex workers, (those whose primary job was serving beer in a beer garden, or providing escort services at karaoke lounges). Because of the law, sex workers in Cambodia have become hidden and shifted away from brothels toward other entertainment jobs such as beer promotion, beer garden waitressing, and karaoke hostessing. Currently, the EW label is used to define women who exchange sexual services for money or goods as income-generation on top of entertainment job [12, 16]. According to the latest national Behavioral Surveillance Survey (BSS) 2010, about 85 % of EWs had reported commercial sexual activities, and more than 79 % of them used to work in a brothel before moving to work in their current type of entertainment job [12].

Traditionally, HIV prevention efforts have focused on consistent condom use as dual protection for HIV, sexually transmitted infections (STIs) and unwanted pregnancies. In Cambodia, though 90 % of EWs reported high consistent condom usage with clients, there was lower and less (<50 %) consistent condom use reported with their “sweethearts”, defined as the partner from a *non-commercial, non-marital sexual relationship that possesses a certain degree of affection and trust* [11]. Moreover, a relatively high number of EWs reported sexual encounters with the latter. This reflects a high risk of unwanted pregnancies and consequently a high abortion rate among these women [11, 17].

These reproductive health (RH) issues (i.e. infrequent use of contraceptives, unintended pregnancy, and abortion) among EWs pose an additional public health concern for HIV programs. To date, this critical linkage between HIV and RH services has been much discussed and prioritised in terms of global policy [18, 19] but still remains weak in terms of country-level implementation, coordination, monitoring, evaluation, and resource allocation [20, 21] especially among those most at risk. For example, issues of unintended pregnancy, contraceptives and access to safe abortion services among EWs have not been integrated well into HIV prevention and treatment programs and/or effectively operated in most developing countries, including Cambodia [10, 22]. In 2009, recognizing that EWs are exposed to multiple risk behaviors and overlapping risk activities and require contraceptive services and access to safe abortion, The National Center for HIV/AIDS, Dermatology and STIs (NCHADS) in collaboration with non-governmental organizations (NGOs) and funding partners revised the standard operating procedure (SOP) for continuum of prevention to care and treatment for most of the at risk population including EWs to include linkage and integration to RH with the aim of maximizing the efficiency of service delivery [16]. Since then, that SOP has been implemented with the focus on the integration of RH services including provision of contraceptives and access to safe abortion services, STI screening and treatment, and referral services (i.e. family planning services and HIV voluntary counselling and testing) [16, 23]. Yet there has been little research on RH characteristics and socio-behavioral factors related to abortion among EWs except a study describing the contraceptive needs of brothel-based sex workers [24]. Much research has been focused on HIV/STI epidemiology and related socio-behavioral determinants [11, 25, 26] but not much on RH including abortion of high risk women. This leaves a major gap in understanding high risk women’s RH needs in addition to what is provided by HIV programs since the implementation of the integration of RH in the HIV prevention and care guidelines among EWs. Therefore, in this paper, we examine socio-behavioral characteristics, and RH practices associated with abortion among Cambodian EWs.

## Methods

### Study setting

In September 2012, a survey was conducted in Siem Reap, Battambang provinces and the capital city Phnom Penh, Cambodia among 595 EWs as part of the mid-term review of Sustainable Action against HIV/AIDS in the Community (SAHACOM) project conducted by KHANA (one of the largest local NGOs working on HIV and RH in Cambodia). The three survey sites represent Cambodia’s most populous areas and the socio-economic hub and touristic sites of the country. According to NCHADS, the estimated number of EWs in Siem Reap, Battambang and Phnom Penh accounts for about 74 % of the total estimated 35,000 EWs in the whole country (*Behavioral Change Communication Unit, NCHADS)*.

### Sampling strategy

The survey sample was collected using two-stage cluster sampling. At the first stage, clusters defined as entertainment establishments (i.e. beer gardens or karaoke bars where EWs worked) were randomly selected with equal probability. At the second stage, all EWs working in the selected establishments were invited for an interview after undergoing informed consent. Using this two-stage sampling approach, randomly selected clusters of different sizes and taking all women in the selected clusters produced a self-weighted sample in each province [11]. All clusters and the number of women in each cluster were listed (as a sampling frame) by the local NGOs and network in collaboration with the respective Provincial AIDS Unit. Co-ordination and administrative support were provided by the local community-based network.

### Data collection

Interviews were conducted face to face by trained female interviewers who were university students under the supervision of supervisors with fieldwork experience. All interviewers participated in a three-day training on interviewer technique, privacy, confidentiality, and questionnaire practices. The questionnaire included socio-demographic information, risk behaviors (i.e. number of partners, condom use), sexual and reproductive information and choices of family planning (FP) method. The close-ended questionnaire was developed based on the national BSS questionnaire, which had been pre-tested and validated many times since implemented in 1997 as part of the national surveillance program. Additional questions were added, based on the objectives and indicators of the SAHACOM review such as awareness about the project implementation, access to outreach and peer education services. The questionnaires were pretested during the interviewer’s training. Verbal informed consent was obtained prior to the interview, and the anonymity and privacy of all study participants were guaranteed. The study protocol was approved by Cambodia’s National Ethics Committee for Health Research at the Ministry of Health.

### Statistical analysis

Data were coded and entered into databases using Epi-Data 3 (Odense, Denmark), and analysis was performed using STATA 12 (Stata Corporation, Texas, USA). All analysis was performed using survey command taking into account the sampling weight of provincial differences of EWs’ population in these provinces and cities although the sampling design was self-weighted within each province [27].

Descriptive statistics were performed to describe all variables in terms of frequency, mean, median, and range. Cross tabulations were performed between variables of interest. The outcome variable “*EW abortion” was defined as report of an abortion while the woman worked as EWs, and self report of ever having at least one abortion*. In univariate and multivariate logistic regression analyses, only women who reported being sexually active (i.e. age reported of first sexual activities) were included in the analysis. Chi-square tests, univariate and multivariate logistic regression were used to assess factors independently associated with “*EW abortion”*. All variables included in the multivariate model were selected based on the research interest and covariates, which were significantly associated with the *EW abortion* at the *p*-value <0.20 in univariate logistic regression [28]. Initially, there were eight covariates: age group, marital status, report of living with whom, duration working as EWs, categories of women’s jobs, and number of male sexual partners, receiving RH information, and the use of different types of FP methods in the past 12 months. However, in the final model, only six covariates (age group, report of living with whom, duration working as EW, category of EW job, number of sexual partners, and receiving RH information) were kept. Associations with a *p*-value <0.05 were considered statistically significant.

## Results

### Socio-demographic characteristics

The refusal rate in this survey was less than 1 %. Of 595 EWs, most were karaoke workers (48.8 %) followed by beer promoters and beer garden waitresses (24.4 %), restaurant workers (18.7 %), masseuses and hairdressers (each <5 %). The mean age of women was 24.5 years old (Median = 24 years), with a range of 15 to 45 years of age and 57 % were 24 years or younger. The EWs had received about 6.5 years of schooling (Median = 7 years), with 6.4 % reporting no education at all. More than 70 % of women were either unmarried or divorced (Table 1).

**Table 1:** Socio-demographic characteristics of entertainment workers

Characteristics		Total (*N* = 595)	
		Frequency	%
Job categories of entertainment workers			
	Beer promotion and beer garden women	128	24.4
	Restaurant, hair cutter and masseuse	159	26.8
	Karaoke women	308	48.8
Age group in years			
	15–19	93	15.2
	20–24	246	41.4
	25–29	173	29.4
	30–45	83	14.0
Education level			
	0–6 years	270	44.8
	7–9 years	232	39.2
	≥10 years	93	16.0
Marital status			
	Unmarried	260	43.4
	Currently married, cohabitated	156	26.3
	Divorced/window	179	30.3
Level of monthly income			
	≤ 120 USD	192	32.6
	121–250 USD	281	47.5
	> 250 USD	120	19.9
Currently living with			
	Parents and relatives	253	43.5
	Spouses and cohabitated	142	24.0
	Friends in rented rooms and alone	200	32.5
Duration of work as entertainment workers			
	< 1 year	265	44.5
	1–2 years	169	28.6
	> 2 years	161	26.9

EWs reporting earnings of about USD210 per month (Median = USD160); 47.5 % of the women earned between USD120 and USD250, whilst 6 % earned only about USD60 per month. Most EWs reported currently living with parents and relatives (43 %), followed by those living with friends in rented rooms or alone (32.5 %), with spouse or sexual partner (24 %). The mean length of time the women had worked as EWs was about two years (Median = 12 months).

### Sexual partners, condom use and reproductive health characteristics

Three-quarters of EWs were sexually active, measured by the percent of women who reported their first age sexual activity. Most sexually active EWs were masseuses (92.8 %, *n* = 47), beer promoters (91.9 %, *n* = 74), and karaoke hostesses (78.4 %, *n* = 308) (Table 2). The average age at first sexual intercourse was 19.4 years old (Median = 19 years), which reflects women’s average age when first married (19.7 years old). Of the 60 % of EWs reporting a sweetheart in the past year, close to 85 % reported having sex with them in the past three months, of which 35 % reported always using a condom (Table 2).

**Table 2:** Sexual behavior and reproductive health characteristics among entertainment workers

Characteristics		Total (*N* = 595)	
		Frequency	%
Sexually active women based on report of first age sexual activities		450	75.0
Sexually active women among different types of EW^a^			
	Masseuses in massage parlors, *n* = 47	43	92.8
	Beer promotion women, *n* = 74	67	91.9
	Karaoke women, *n* = 308	241	78.4
	Restaurant workers, *n* = 99	65	60.7
	Beer garden women, *n* = 54	27	53.1
	Hair cutters in beauty salon, *n* = 13	7	53.8
		Total (*N* = 450)^b^	
Sweetheart in past 12 months		272	60.4
Sex with sweetheart in past 3 months (*n* = 272)		229	84.0
Always use condom with sweetheart in the past 3 months (*n* = 220)		75	35.0
Sex with clients in the past 12 months		167	37.7
Always use condom with clients in the past month (*n* = 120)		103	84.0
Number of male sexual partners in past 12 months (*n* = 394)			
	One partner	220	55.5
	2–7 partners	107	27.7
	> 7 partners	67	16.8
Received information about SRH in the past 12 months		401	89.5
Referred to SRH services in the past 12 months		135	31.5
Referred to family planning services in the past 12 months		71	18.1
Contraceptive methods used in the past 12 months			
	Nothing	141	29.6
	Condom	172	38.2
	Withdrawal	56	13.6
	Pill	45	10.0
	Others (Injection, calendar, abortion)	25	8.6
Abortion while working as entertainment workers		122	28.5
Time since last abortion (*n* = 196)			
	≤ 6 months	75	38.3
	7–23 months	52	26.5
	≥ 24 months	69	35.2
Places where EWs sought last abortion (*n* = 196)			
	Private clinic	90	47.0
	Self-abortion by medicine from pharmacies	65	30.5
	Public and NGO clinics	41	22.5

^a^ EWs: entertainment workers; ^b^ among sexually active entertainment workers only

Close to 38 % of EWs reported having had sex with clients in the past 12 months; karaoke hostesses and beer promoters accounted for the highest proportion of those having had paid sex (43–44 %); 84 % of the women reported always using a condom in the past month. The report of condom use with last client was as high as 90 %. On average, EWs had had 5.5 different clients in the past 12 months; 45 % of women reported more than one partner in the past 12 months (Table 2). Only 3.2 % of women reported clients who refused to use condoms (i.e. clients offering extra money not to use a condom, threatening—either verbally or physically, or being under the influence of alcohol or drugs).

Almost all EWs (90 %) reported having received information about RH and FP in the past 12 months. One third of the women reported not using any FP methods with partners, 38.2 % had used condoms as their contraceptive method, which was followed by the withdrawal, pill and other methods (Table 2). Of sexually active women, 35 % had experienced at least one pregnancy while working as an EW. Furthermore, 28.5 % of the women reported having experienced at least one abortion while working as an EW. Of these, 38.3 % had experienced at least one abortion in the past six months. EWs reported most of the abortions were performed at a private clinic (47 %), but many also reported self-abortion using medicine (30.3 %) bought from pharmacies (Table 2).

### Socio-demographic and behavioral factors associated with abortions

Results of univariate analysis for the socio-demographic and behavioral factors associated with *“EW abortions”* are presented in Table 3. Factors found to be significantly associated with abortion included age group 25–29 (with age ≤ 24 as a referent group), (Odds ratio [OR] = 1.7 with 95 % confidence interval [CI]: 1.0–2.7), currently married or cohabitating partner (OR = 2.1, 95 % CI: 1.3–3.5), living with spouse or cohabitated partner (OR = 2.5, 95 % CI: 1.4–4.2), more than seven sexual partners in the past 12 months (OR = 3.3, 95 % CI: 1.8–5.9), and working as a karaoke hostess (OR = 2.4, 95 % CI: 1.3–4.3). We also found that the longer women had worked, the higher the risk of having abortions; 2.9 times higher when working as EW longer than one year (95 % CI: 1.6–5.2), and 2.7 times higher when working as EW longer than two years (95 % CI: 2.7–8.3). Additionally, covariates such as having received RH information and using different FP methods were found to be significantly associated with reporting abortion.

**Table 3:** Factors associated with abortion among entertainment workers: univariate logistic regression

Characteristics		Total *N =* 450^a^		*P* value
		No. (% abortion)	OR (95 % CI)^b^	
Age group (in years)				
	≤ 24	48 (24.1)	Referent	
	25–29	52 (34.6)	1.7 (1.0–2.7)	0.035
	≥ 30	22 (27.8)	1.2 (0.7–2.3)	0.524
Education level				
	≥ 10 years	13 (24.1)	Referent	
	7–9 years	53 (32.6)	1.5 (0.7–3.1)	0.252
	0–6 years	56 (26.7)	1.1 (0.6–2.3)	0.705
Marital status				
	Divorced and widow	33 (21.7)	Referent	
	Unmarried	26 (25.0)	1.2 (0.7–2.2)	0.546
	Currently married, cohabitated	63 (36.7)	2.1 (1.3–3.5)	0.004
Level of monthly income				
	≤ 120 USD	30 (26.1)	Referent	
	121–250 USD	55 (26.0)	1.0 (0.6–1.7)	0.986
	> 250 USD	37 (36.8)	1.7 (0.9–3.0)	0.100
Current housing status				
	Friends and alone	32 (21.2)	Referent	
	Parents and relatives	35 (25.1)	1.2 (0.7–2.2)	0.446
	Spouses or cohabitated partners	55 (40.0)	2.5 (1.4–4.2)	0.001
Duration of work as entertainment worker				
	< 1 year	24 (14.0)	Referent	
	1–2 years	40 (32.1)	2.9 (1.6–5.2)	<0.001
	> 2 years	58 (43.4)	4.7 (2.7–8.3)	<0.001
Job categories of EWs				
	Restaurant, hair cutter, masseuse	19 (16.6)	Referent	
	Beer promotion and beer garden women	30 (32.8)	2.5 (1.2–4.9)	0.010
	Karaoke women	73 (32.1)	2.4 (1.3–4.3)	0.005
No. of men had sex with in past 12 months				
	1 partner	55 (25.6)	Referent	
	2–7 partners	30 (29.3)	1.2 (0.7–2.0)	0.509
	> 7 partners	31 (53.1)	3.3 (1.8–5.9)	<0.001
Received information on SRH in the past 12 months				
	No	7 (13.7)	Referent	
	Yes	115 (30.2)	2.7 (1.2–6.4)	0.020
Referred to SRH services in the past 12 months				
	No	80 (26.7)	Referent	
	Yes	42 (32.8)	1.3 (0.8–2.1)	0.208
FP methods EWs used in the past 12 months				
	Nothing	24 (17.8)	Referent	
	Condom	49 (30.1)	2.0 (1.2–3.6)	0.015
	IUD/pill/Injection	23 (34.1)	2.4 (1.2–4.7)	0.013
	Withdrawal/calendar	26 (38.1)	2.8 (1.4–5.7)	0.003

^a^ Numbers may not sum to total of 450 for some variables due to missing data

^b^ Odds ratio and 95 % confidence interval

In the final multivariate model (Table 4) the following were significantly associated with report of abortions while an EW: age-group 25–29 (OR = 2.2, 95 % CI: 1.2–4.0), living with spouse or cohabitated partner (OR = 2.2, 95 % CI: 1.1–4.2), longer duration working as EW (> one year) (OR = 3.0, 95 % CI: 1.6–5.7), and longer than two years (OR = 4.8, 95 % CI: 2.5–9.2), working as karaoke hostess (OR = 2.2, 95 % CI: 1.1–4.4), and reporting sexual partners > 7 in the past 12 months (OR = 4.4, 95 % CI: 2.2–8.7). Reports of receiving SRH information were not significantly associated with abortion.

**Table 4:** Factors associated with abortion among EWs: multivariate logistic regression

Characteristics		Total, *N =* 394^a^	*P* value
		AOR (95 % CI)^b^	
Age group (in years)			
	≤ 24	Referent	
	25–29	2.2 (1.2–4.0)	0.01
	≥ 30	1.1 (0.5–2.5)	0.86
EWs reported currently live with			
	Friends and alone	Referent	
	Parents and relatives	1.0 (0.5–1.9)	0.92
	Spouse or cohabitated partner	2.2 (1.1–4.2)	0.02
Duration of work as entertainment worker			
	< 1 year	Referent	
	1–2 years	3.0 (1.6–5.7)	0.001
	> 2 years	4.8 (2.5–9.2)	<0.001
Job category			
	Restaurant, hair cutter, masseuse	Referent	
	Beer promotion and beer garden	1.6 (0.7–3.6)	0.24
	Karaoke	2.2 (1.1–4.4)	0.02
No. of men having sex with in past 12 months			
	1 partner	Referent	
	2–7 partners	1.6 (0.9–3.0)	0.11
	> 7 partners	4.4 (2.2–8.7)	<0.001

^a^ *N =* 393 in the final multivariate logistic regression due to missing data in some variables

^b^ Adjusted odds ratio and 95 % confidence interval

## Discussion

Our findings demonstrate that three quarters of the entertainment workers in our survey in Cambodia’s three most populous provinces were young and sexually active with a high proportion reporting history of recent abortion. In particular, the highest risk of having an abortion was found among women aged 25–29 years, and those currently living with spouses or cohabitated partners. The longer duration work as an EW clearly puts women at higher risk of unprotected sex resulting in higher reports of abortion. Finally, the risk of having an abortion was found to be linked to a specific type of entertainment job (i.e. working in karaoke establishments). This provides RH services a focus of whom among EWs to prioritize.

The study finding that not all EWs are sexually active, the exceptions being the masseuses, beer promoters and karaoke hostesses also helps provide programs focus. Given Cambodia’s recent budget constraints, this provides guidance on which categories of EWs contain those most sexually active and avoids targeting the whole EW population. However, non-sexually active women should be provided with behavioral change communication messages about HIV and RH before they become at risk of HIV infection and RH issues.

Our findings reveal nearly one third of the EWs experienced at least one abortion while working as EWs. This high rate of recent abortion among these women suggests that condom use alone as the dual protection against HIV and pregnancy is not effective in preventing unintended pregnancies, especially with non-paid partners (i.e. sweethearts). Non-barrier methods should be introduced given the low condom use and low use of other contraceptive methods. When compared with reports from women of reproductive age in the general population, the EWs are experiencing very high rates of abortions, especially those aged 25–29. Interestingly, data from Laos and Russia shows the risk of abortion among EWs increased with age [9, 24]. Our further analysis found that more women aged 25 years and older reported a having sweethearts than younger women, increasing their likelihood of an unintended pregnancy because of the low condom and contraceptive use with such partners. Therefore, integrated interventions should be focused on RH referral services beyond always condom use to contraceptive and safe abortion services, as was recommended in the HIV and RH integrated SOP.

EW women who reported living with spouses or cohabitated partners also had an increased risk of abortion. Anecdotal evidence suggests that many sweethearts of EWs are previous clients [16, 29]. Over time, their regular relationship had become intimate and transformed a client into a regular client and then sweethearts and eventually, to some extent, many eventually become EWs’ cohabitating partner or spouse. This relationship pattern represents a challenge for an HIV prevention program and suggests an urgent need for practical, feasible and realistic RH services, which could be integrated as part of the HIV integrated program. As shown in the results, consistent condom use with a sweetheart was as low as 35 % and this was confirmed in other reports [11, 12, 24]. Over the years, consistent condom use among sex workers or EWs with their sweethearts has never exceeded 50 % although repeated efforts have been made to address the issue [11]. Condom use is seen to reduce intimacy and reflects a lack of trust. Working in entertainment jobs while maintaining an ongoing sexual partnership seems to put these women at the greatest risk of abortion, as negotiating an effective and consistent approach to family planning may be particularly challenging within the established partnership.

While many women working in entertainment establishments are exposed to health education about condom use for HIV/STI prevention through mass media outreach and peer education programs, there is little discussion of broader RH issues apart from referral for STI screening and treatment. Our findings suggest that in addition to condom promotion, specific RH needs including access to safe abortion services, avoiding unintended pregnancy, and choice of contraceptives, are of critical importance for women working in entertainment jobs and establishments [22]. In the past, abortions have been widely used by EWs voluntarily to deal with unintended pregnancies although it is perceived as both risky and costly [10]. As shown in our findings, contraceptive methods used were still unacceptably low, including condom use with intimate partners. This suggests that intimacy, trust, love, social support and safer financial security from their cohabitated partner or sweethearts may prohibit use of contraception posing further hurdles for the HIV integrated services.

Recognizing that EW’s practice multiple risk behaviors and lack of access to contraceptive and safe abortion, the SOP for HIV prevention to care with an integration to RH has been implemented [16]. However, no research has been conducted to assess the extent to which this integrated SOP implementation is effective and feasible. Furthermore, anecdotal evidence suggests that although the HIV and RH integrated SOP has been implemented, erratic activities have been documented across provinces due to a number of reasons, such as lack of motivation for local HIV staff, referrals issues to RH clinics, and underfunding (about 90 % of the HIV and RH activities have been supported through external funding). This was also supported by the findings from other settings, where the delivery of HIV and RH programs always face challenges in terms of policy priority, reporting, monitoring and evaluation system coordination between stakeholders in HIV and RH programs, and the underfunded RH [21, 30]. These anecdotal reports of program difficulties along with the clear lack of utilization of broader contraceptive services reported by EWs in our survey, suggests Cambodia’s integrated HIV/RH program has a long way to go to reaching those most at need.

The link between duration of work in an entertainment job and increased risk of abortion suggests a cumulative exposure to health challenges, to HIV/STI and RH issues, and especially unsafe abortions. This finding was confirmed in many studies in different settings [9, 24]. An example is work as a karaoke hostess that was associated with a higher risk of abortion than work in other entertainment categories. While one third of the women working as karaoke hostess report an abortion during their time in entertainment work, only about 5 % of Cambodian women in the general population report an abortion in past 5 years [6]. Therefore, the elevated percent of women working in karaoke who have an abortion may reflect the nature of this working environment where women have to entertain clients with songs, escort services and alcohol, and ultimately the negotiated sexual services. Such commercials sexual encounters while also working as a “performer” may prove more challenging for women. We also found that karaoke hostesses earned twice as much as other women (data not shown) also suggesting selling sex or these women may operate on different terms than for other women working in entertainment Finally, another study found karaoke hostesses report more clients and more paid sex compared to beer promoters [31] again pointing to their elevated risks of high risk sexual exposures.

The study has faced a number of limitations. The first concern relates to the social desirability bias [32] embedded in asking about personal and sensitive information, including past sexual practices, condom use and abortion in a face to face interview. EWs who have been frequently exposed to outreach and peer education programs may intend to give positive and desirable responses to interviewers; however, this type of bias may be reduced to a minimum through the use of the national validated questionnaire and training of female interviewers in dealing with such sensitive and personal information. Additionally, the fieldwork was undertaken under close supervision to ensure the privacy, confidentiality and anonymity of the participants. The second concern might be that the RH terminology might not have been clear in the questionnaire since it was originally designed to collect information about the HIV prevention and care program. However, concern about these limitations is reduced because we found that our findings were consistently in line with the results of the past studies [9, 11, 24].

## Conclusions

This study highlights a relatively high proportion of EWs reporting abortion. While the vulnerability of the young women who work in this industry for HIV/STI acquisition has been clearly established, their broader sexual and RH needs have gone largely unrecognized and have not been prioritized. The HIV and RH integrated program initiatives were introduced in 2009 by the HIV prevention community in Cambodia; however, challenges for these women of multiple risks from professional as well as personal partners are profound. For these young women the context of high risk sex intertwined with development of intimate partnerships points to the need for multipurpose technologies that combine pregnancy and HIV/STI prevention in a single method. While such methods are in development, there is an urgent need for practical programmatic approaches including expanded contraceptive and abortion care services to help to improve the RH of these young women.
